# Taming Variability in T-Cell Mechanosensing

**DOI:** 10.3390/cells14030203

**Published:** 2025-01-30

**Authors:** Paula J. Schultheiss, Aarya Pulkundwar, Wangqi Li, Lance C. Kam

**Affiliations:** 1 Department of Biomedical Engineering, Columbia University, New York, NY 10027, USA; pjs2199@columbia.edu (P.J.S.); ap4486@columbia.edu (A.P.); 2 Department of Computer Science, Columbia University, New York, NY 10027, USA; wl3019@columbia.edu; 3 Department of Medicine, Columbia University, New York, NY 10027, USA

**Keywords:** CAR T-cell therapies, biomaterials, mechanosensing, immunotherapy

## Abstract

A central step in T-cell immunotherapy is the expansion of a starting population into therapeutically potent numbers of these “living drugs”. This process can be enhanced by replacing the mechanically stiff materials used for activation with softer counterparts. However, this mechanosensitive expansion response varies between individuals, impeding the full deployment of potential cell immunotherapy. This report identifies the sources of this variability, ultimately improving the reliability of T-cell expansion. T cells from a cohort of healthy donors were phenotypically characterized, activated, and expanded in vitro on soft and hard substrates, capturing and quantifying a wide range of mechanosensing responses. An analysis of expansion against demographic and phenotypic features correlated mechanosensing with the percentage of effector T cells (T_Eff_s) in the starting population. Depletion experiments confirmed that T_Eff_s mediate mechanosensitive expansion but also suggest that these cells are not responsible for large-scale cell production. Instead, population-level expansion results from interactions between T-cell subtypes. By providing a framework and experimental approach to understanding donor variability, the results of this study will improve the success and reliability of T-cell immunotherapy.

## 1. Introduction

T cells are key players of the adaptive immune response, fighting a wide range of pathogens and pathogenic cells. These cells also have tremendous potential as “living drugs” against a range of diseases, seen most prominently in the development of adoptive cellular therapy (ACT) targeting cancer [1,2,3]. A central step across many variants of this approach is the ex vivo activation and expansion of a smaller starting population to yield clinically significant numbers of T cells. Variability in this production step remains a barrier to effective deployment of T-cell-based ACT, particularly when applied to chronic cancers in which T cells exhibit exhaustion [4,5,6].

An emerging approach toward improving ex vivo expansion is to modulate the mechanical stiffness of materials used to activate cells and initiate expansion, leveraging the ability of T cells to carry out mechanosensing particularly in the context of disease [7]. In particular, the stiffness of tumors differs from that of healthy tissue, suggesting that local mechanical and topographical properties of the in vivo microenvironment may direct immune cell response, including the exclusion of cells from malignant tissue [8,9]. Mimicking such features in biomaterials is emerging as a powerful approach to tuning immunotherapy [10,11]. For ex vivo expansion, T cells are activated by engaging the cells’ surface receptors, CD3 and CD28, with antibodies attached to a solid support (such as the Dynabead or TransAct platforms from Thermo Fisher and Miltenyi), providing activation and costimulation signaling, respectively. Surprisingly, replacing the stiff materials used in conventional activation systems to support α-CD3 and α-CD28 with softer counterparts produced almost an order of magnitude more cells per round of expansion [12,13,14]. These materials even rescued the expansion of cells from Chronic Lymphocytic Leukemia patients, which often express features of exhaustion and low proliferation, posing challenges for cellular immunotherapy [15]. However, the degree to which a population of T cells responded to substrate stiffness varied dramatically between donors, which is a new facet of the variation in overall expansion seen between individual donors that has only recently begun to be addressed [16,17].

This report sheds light on the sources of donor-to-donor variability in the mechanosensitive expansion of T cells. We analyzed mechanosensitive expansion as a function of multiple demographic, biomarker, and subtype characteristics, identifying a specific feature associated with this response. By uncovering the mechanisms behind donor variability, we aim to inform the design of biomaterials that optimize mechanosensitive T-cell expansion and mitigate inter-donor variability. This framework can be directly applied to other approaches seeking to improve T-cell expansion, ultimately broadening patient access and enhancing therapeutic outcomes.

## 2. Materials and Methods

PDMS preparation. Polydimethylsiloxane (PDMS) substrates were created using a Sylgard 184 (Dow Corning, Midland, MI, USA) platform following previously described techniques [13,18]. Mechanically “Hard” substrates were prepared by mixing the curing agent and elastomer base components at a standard 1:10 (*w*/*w*) ratio, while “Soft” substrates used a 1:50 ratio. Uncured mixes of these components were centrifuged at 3000× *g* for 3 min, and then distributed into wells of 24-well culture plates. The PDMS was degassed under vacuum and then cured for 18 at 65 °C. PDMS substrates were treated with 250 µg/mL of gentamycin for 2 h, washed extensively, and then coated with 1 µg/mL of biotin-conjugated goat anti-mouse IgG antibody (BioLegend, Way San Diego, CA, USA) at room temperature. To ensure even distribution of the antibodies across the PDMS surfaces, the culture plates were placed on an orbital shaker at 140 rpm for 2 h. Substrates were blocked for 2 h at room temperature using 5% bovine serum albumin (BSA, Sigma-Aldrich, Burlington, MA, USA), and then coated with 50 µg/mL of a 1:4 mixture of 10 µg/mL α-CD3 (clone OKT3, BioXCell, Dartmouth College, NH, USA) and 40 µg/mL α-CD28 (clone 9.3, BioXCell) antibodies for 2 h at room temperature or overnight at 4 °C. These conditions were adopted from our previous studies [7,11] for consistency.

PDMS stiffness characterization. The elastic properties (Young’s modulus) of PDMS substrates were characterized using an Instron 5848 Microtester (Instron, Norwood, MA, USA) with a 12 mm diameter flat cylindrical head. The substrates were indented in three steps with 0.1 mm depth each. The speed of indentation was 0.1 mm/s. Between steps, the material was allowed to relax for 10 s.

ELISA. The ability of antibody-presenting PDMS surfaces to engage their targets was compared using an ELISA assay conducted as follows: Initially, PDMS substrates were coated with 1 µg/mL of biotin-conjugated goat anti-mouse IgG for 2 h, followed by three washes with PBS. A secondary mouse anti-rabbit IgG antibody at a concentration of 3 µg/mL, diluted in 5% BSA, was then added for 2 h at room temperature. After washing, the plates were coated overnight at 4 °C with 1 µg/mL rabbit anti-chicken horseradish peroxidase (HRP) antibody, diluted in 5% BSA. Following a final set of three PBS washes, 1:1000 TMB substrate was added and incubated for 5 min in the dark without agitation, ensuring complete coverage of the wells’ surface. The reaction was stopped by adding 1 M H2SO4 in the same order as the TMB application, and absorbance was measured at 450 nm.

Cell isolation and culture. Primary human CD3^+^ T cells (including both CD4^+^ and CD8^+^ subtypes) were obtained from leukapheresis preparations from healthy adult donors (New York Blood Center). CD3^+^ T cells were isolated by negative selection using RosetteSep Human T cell enrichment kits (Stem Cell Technology, Vancouver, BC, Canada) followed by density gradient centrifugation with Ficoll-Paque PLUS (GE). Isolated cells were cultured in complete culture media consisting of RPMI 1640 (ThermoFisher, Waltham, MA, USA) supplemented with 10 mM 4-(2-hydroxyethyl)-1-piperazine ethanesulfonic acid (HEPES, Gibco, Waltham, MA USA), 10 mM L-glutamine (Gibco), 10% (*v*/*v*) fetal bovine serum (FBS; Gibco), 0.34% (*v*/*v*) β-mercaptoethanol (Sigma-Aldrich), and 10 mM penicillin-streptomycin (Gibco). Post-isolation, the cells were cryopreserved in complete media containing 40% FBS and 10% dimethyl sulfoxide (DMSO) and stored in liquid nitrogen. Prior to experiments, the cells were thawed and allowed to rest in standard culture conditions (37 °C, 5% CO_2_/95% air) overnight.

T-cell expansion was carried out following established methods [13,14,19]. For the activation of T cells, 1 × 10^6^ mixed T cells in 1 mL of media were either seeded on activating PDMS surfaces or mixed with CD3/CD28 Dynabeads at a 1:1 bead:cell ratio and cultured in wells of 24-well plates under standard cell culture conditions (37 °C, 5% CO_2_/balance Air, 100% humidity). Every second day, starting on day 3 after seeding, the T cells were collected, diluted to 500k cells/mL with fresh media, and reseeded in uncoated tissue culture plates for expansion. Expansions were carried out until the number of cells resulting from an expansion reached a maximum and then began to decrease.

Cell staining and flow cytometry. Phenotypic marker expression was assayed using antibodies against human CD4 (clone OKT4, labelled with Brilliant Violet 510, dilution 1:100), CD8 (clone SK1, APC/Cyanine7, 1:100), CD45RA (clone HI100, Alexa 647, 1:50), CD62L (clone DREG-65, PE-Dazzle 594, 1:25), CD279 (BD, clone EH12.1, RB780, 1:50), CD95 (clone DX2, PE/Cy7, 1:50), CD25 (clone BC96, Pac-Blue, 1:25), and CD137 (BD, clone 4B4-1, BUC737, 1:50), which were all from BioLegend except when specified. Dead cells were stained using a Live/Dead Fixable Green Staining Kit (Invitrogen, Waltham, MA, USA). A total of 2 × 10^5^ cells were labeled in 50 µL with antibodies for 30 min at room temperature in the dark before fixation using a True-Nuclear™ Transcription Factor Buffer Set (BioLegend). Data were collected using a 5 L Aurora (Cytek, Fremont, CA, USA) flow cytometer. Data analysis was carried out using FCS Express V6 (De Novo, Louisville, CO, USA). The gating strategy is shown in Figure S1.

Subtype depletion and reconstitution. Starting T-cell populations with modified subtype profiles were prepared using bead-based purification and reconstitution methods. In particular, populations depleted of Effector (T_Eff_) cells were prepared by first separating CD62L^+^ cells from CD62L^−^ cells using a REAlease^®^ CD62L MicroBead Kit (Miltenyi Biotec, Washington, DC, USA). Next, CD62L^−^ cells were separated into CD45RA^+^ T_Eff_ and CD45RA^−^ Effector Memory (T_EM_) subtypes using CD45RA Microbeads (BioLegend). Finally, the CD62L^+^ and T_EM_ cell fractions were recombined in proportions that mirror the original population’s composition, depleted of T_Eff_ cells. CD62L^+^ vs. CD62L^−^ populations were prepared for additional experiments using only a CD62L MicroBead Kit.

Statistical analysis. Statistical analyses were performed using Prism v8.01 (Graphpad, La Jolla, CA, USA) and Python 3.11. To investigate the underlying structure of the dataset and to identify a compact set of factors explaining the majority of the variance between donors, a principal component analysis (PCA) was conducted using the scikit-learn library in Python. Traditional statistical analyses were conducted using both one-way and two-way Analysis of Variance (ANOVA), accompanied by Tukey’s method for multiple comparisons, utilizing GraphPad Prism version 7 (GraphPad Software). A significance threshold was set at a p-value of less than 0.05. Unless specified differently, all error bars represent standard deviations. Correlation analysis was performed to assess the relationships between different phenotypic variables of T cells and the mechanosensitivity factor (MSF). Pearson’s correlation coefficient was used to quantify the linear correlation between pairs of variables.

## 3. Results

### 3.1. Mechanosensitive Ex Vivo Expansion Varies Across Individuals

**Figure 1:**
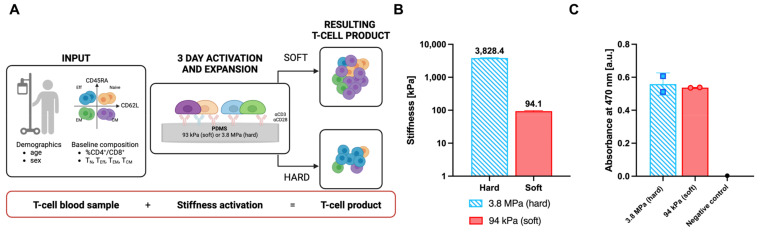
Study overview and substrate characterization. (**A**) Schematic of the experimental design investigating the influence of donor characteristics on the mechanosensitive expansion. T cells were isolated from blood samples and the donors’ T-cell phenotypic composition was characterized. CD3^+^ T cells were activated on PDMS of different stiffnesses and expanded. (**B**,**C**) Characterization of PDMS substrates for T-cell activation. (**B**) Substrates of two distinct stiffnesses (Hard and Soft) were fabricated by varying the cross-linker concentration (n = 5 per condition). Data are mean ± s.d. (**C**). Despite different mechanical stiffnesses, these surfaces showed equal capacity to bind to their antibody targets as demonstrated by measuring their antibody surface concentration with an ELISA (n = 2). Negative control—hard surface, no IgG + TMP substrate.

To capture the variability in T-cell mechanosensing response across a wide range of individuals, we directly compared the expansion of cells from a cohort of 23 healthy donors as a function of substrate stiffness (Figure 1A); while future applications include treatment of diseases, this study focuses on cells independent from the complexities of the disease state as an initial proof-of-concept. These substrates consisted of PDMS elastomer presenting antibodies to CD3 and CD28 (clones OKT3 and 9.3, respectively). Substrate stiffness was modulated by changing the ratio of curing agent to elastomer base [13] and expressed in terms of Young’s Modulus, E. Two specific formulations were chosen to produce Hard (3.8 MPa, 1:10) and Soft (100 kPa, 1:50) substrates (Figure 1B), which were conditions previously shown to capture the T-cell mechanosensing response [13]. Notably, this approach allowed control over substrate stiffness independent of the ability of these surfaces to capture antibody targets (Figure 1C).

**Figure 2:**
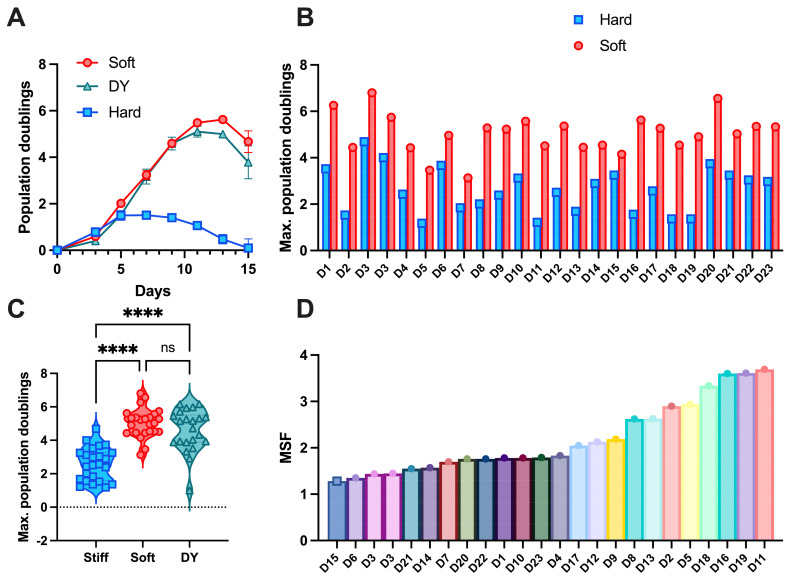
T-cell expansion in response to substrate stiffness is donor-dependent. (**A**) An exemplary expansion profile of T cells activated on substrates of two different stiffnesses (Donor D16). Dynabeads were used as a positive control for activation (n = 2). Data are mean +/− s.d. (**B**) Averaged maximum population doublings of T cells activated on Soft and Hard PDMS. The maximum population doublings for each donor on Soft vs. Hard substrates reveal individual differences in the mechanosensitive response. (**C**) The maximum population doublings on two substrate stiffnesses across all donors and experiments show a significant increase in expansion on Soft surfaces. Statistical significance was determined using a one-way ANOVA followed by a Tukey multiple comparison test across all cells captured for each condition (n = 23). **** *p* < 0.001, ns = non-significant. (**D**) The mechanosensitivity factor (MSF) offers a quantitative measure of the mechanosensitive expansion effect, which varies considerably across donors. For this graph, donors are presented in order of increasing MSF to illustrate the distribution of this readout across individuals.

Primary human CD3^+^ T cells, including both CD4^+^ and CD8^+^ T cells, were activated on these surfaces and expanded over several weeks. A typical expansion time course is shown in Figure 2A, reporting cumulative population doublings over time. In agreement with previous studies [13,18], Soft substrates induced an extended phase of robust, long-term proliferation and more doublings compared to their Hard counterpart. The maximum doublings (MD) reached in each expansion was used as a measure of proliferative potential, allowing comparison across conditions. This pattern of increased expansion following activation on Soft vs. Hard PDMS was seen across all 23 donors (Figure 2B,C). Expansion following activation with Dynabeads was highly variable but closer to that resulting from the Soft surface; notably, the identity and concentration of antibodies presented by Dynabeads are different from those presented by PDMS substrates, complicating direct comparisons between these platforms. As a single, numerical readout allowing comparison of mechanosensing between donors and across experiments, we introduce a mechanosensitivity factor (MSF) calculated as the ratio of maximum doublings obtained on the Soft vs. Hard substrate. Notably, the MSF showed lower variability between replicate expansions of cells from a given donor than the maximal doublings on the Soft surfaces (MSF: avg = 1.37, s.d. = 0.033, s.d./avg = 0.0248; MD_Soft_: avg = 4.16, s.d. = 0.235, s.d./avg = 0.565; n = 20 samples across eight donors), making this a strategic readout for analysis. While the MSF was consistent between experiments for individual donors, this readout varied over three-fold across donors (Figure 2D), revealing the range and individuality of mechanosensing as well as illustrating the challenges facing T-cell manufacture.

### 3.2. Variation in T-Cell Mechanosensing Correlates with T_Eff_ Frequency

**Figure 3:**
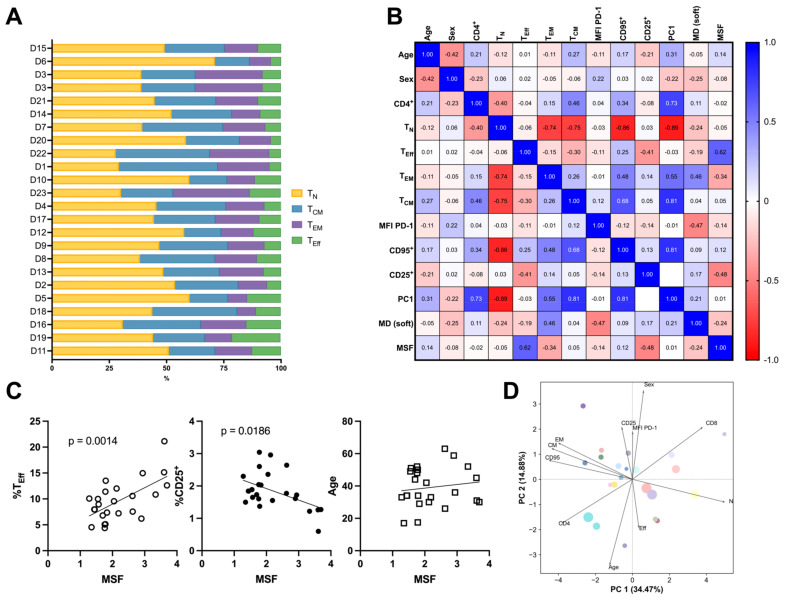
T-cell subtype frequency within bulk CD3^+^ T-cell populations correlates with mechanosensing. (**A**) Frequencies of individual donors’ CD3^+^ T-cell subtypes assessed by flow cytometry. Each donor’s proportion of T_N_, T_CM_, T_EM_, and T_Eff_ cells was profiled before expansion. Frequencies of these subtypes divided further into CD4^+^ vs. CD8^+^ cells are included in Table S1. Donors are presented in order of increasing MSF from top to bottom of the *y*-axis. (**B**) Pearson correlation analysis between demographic and subtype features and expansion outputs (n = 23). The correlation analysis revealed that the percentage of CD3^+^ T_Eff_s positively correlates with the MSF. MFI = median fluorescence intensity, MD = maximum doublings, MSF = mechanosensitivity factor. (**C**) Linear regression analysis for select features and the MSF. *p* values are included when less than or equal to 0.05. (**D**) Biplot of PCA analysis across demographic and subtype factors. Symbols are color-coded by donor following Figure 2D, while the symbol size indicates the MSF.

We next aimed to identify features of a donor’s T-cell sample that contribute to and explain the variability in mechanosensitive expansion shown in Figure 2D. Candidate features (Table S1) included demographic data of donor sex and age as well as the subtype composition of each donor’s cells, measured before expansion (a “baseline” profile of subtypes). Subtypes included Naive (T_N_), Central Memory (T_CM_), Effector Memory (T_EM_), and Effector (T_Eff_), which showed significant variation between donors, both when combined (Figure 3A) and further separated into CD4^+^ and CD8^+^ populations (Table S1, Figure S2). The exhaustion marker PD-1, activation marker CD95, and regulatory T-cell marker CD25 were also assessed. We began with a correlation analysis between all pairs of features and measures of expansion, which revealed that T_Eff_ and CD25^+^ cells in the total CD3^+^ population had the strongest correlations with the MSF (Figure 3B and Figure S3). Notably, CD8^+^ cells within the T_Eff_ subtype showed strong correlation with the MSF (Figure S3) while CD4^+^ cells did not, illustrating an effect of granular subtyping (Figure S3). However, the total T_Eff_subtype (CD4^+^ + CD8^+^) showed the highest correlation with the MSF, suggesting a stronger impact of this combined group with regards to mechanosensing. Plots of T_Eff_ and CD25 vs. MSF showed linear associations with strong significance (*p* < 0.001 and *p* < 0.02, respectively) and no systematic residuals, suggesting that higher-order expressions would not improve the fit (Figure 3C). Regression of age vs. MSF revealed neither a significant linear effect nor that a higher-order relationship would be appropriate (Figure 3C). Similarly, no effect of sex was observed by this method. Permutation analysis examining the MSF as a function of sex (male vs. female) and age (stratified into those above and below the average age of 40) revealed no effect (*p* < 0.87 and *p* < 0.77, respectively), supporting the result of correlation analysis with a method that is less sensitive to sample size. Approaches to combine factors to explain variation in MSF were also tested. In particular, Principal Component Analysis (PCA) applied to demographic and subtype data revealed only moderate dimensionality reduction within the input factors (Figure 3D and Figure S4). In addition, the MSF, encoded by the size of each data point in Figure 3D, showed little correlation with PC1/PC2 (Figure S5). Together, these results suggest that T-cell subsets exhibit different degrees of mechanosensing and that focusing on a specific group (most notably T_Eff_) brings a productive understanding of how complex populations of T cells respond to substrate stiffness.

### 3.3. T_Eff_ Cells Mediate Mechanosensing

**Figure 4:**
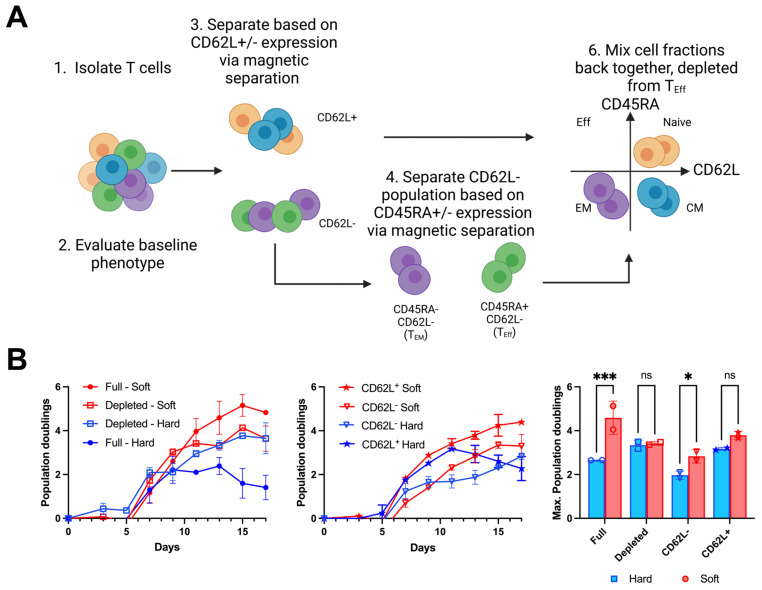
T_Eff_ cells modulate population-level mechanosensing. (**A**) Schematic of the experimental design to unravel CD3^+^ T_Eff_ cells’ contribution to mechanosensitive expansion. Bead-based separation and reconstitution methods were used to modulate the subtype composition of starting cell populations by depleting T_Eff_ cells. (**B**) Exemplary expansion profiles of Full and modified T-cell populations from the same donor. Depletion of T_Eff_ cells abolishes the differences in expansion between Soft and Hard surfaces. A comparison of maximum doublings emphasizes the role of T_Eff_s in modulating expansion and driving mechanosensitivity (n = 2 per condition). * *p* < 0.05, *** *p* < 0.001, ns = non-significant. Data are mean +/− s.d across replicate samples for a representative experiment.

Next, we tested whether T_Eff_ cells do mediate mechanosensing by measuring the MSF for starting populations depleted of specific subtypes. In particular, bead-based purification and reconstitution methods were used to prepare T_Eff_-depleted populations for comparison with Full counterparts from the same donor (D16, Figure S6). Depleting T_Eff_ cells abrogated the mechanosensitivity seen for the full, complete population (Figure 4B). By contrast, populations of CD62L^−^ cells (a mix of T_EM_ and T_Eff_ subtypes) retained mechanosensing; these two results identify a key role of T_Eff_ cells in population-level mechanosensing. It is noted that expansion of CD62L^−^ cells was lower than that of either the Full or T_Eff_-depleted groups, indicating that the overall production of cells was not associated with T_Eff_ or T_EM_ cells. Rather, T_Eff_-depleted and CD62L^+^ populations showed long-term expansion that was higher than the CD62L^−^ preparation, suggesting that overall population growth is associated with non-T_Eff_ subtypes, in particular T_N_ cells, which comprise the greatest fraction of cells for most of the donors (Figure 3A) and are associated with high proliferation. Together, these results suggest a complex interdependence between cell subtypes, where T_Eff_s provide mechanosensing that drives expansion of T_N_ and possibly T_CM_ subtypes.

## 4. Discussion

An individual’s immune response arises from a complex interplay of genetic, environmental, and phenotypic factors, resulting in significant variability in cellular functionality [16,20,21]. Unlike molecular drugs, where quality can be ensured by maintaining consistent raw materials, this inherent variability complicates adoptive T-cell therapies currently under development, affecting a range of outcomes from the success of ex vivo expansion to standardization of the final product and in vivo efficacy [22,23]. The ability to identify factors of a starting cell population that predict its response to specific manufacturing parameters would dramatically improve the consistency and impact of cellular immunotherapy [24,25].

This study develops such an understanding in the context of leveraging T-cell mechanosensing toward improved ex vivo expansion. Specifically, we showed that the simple measure of the percentage of T_Eff_ cells in a starting population reflects the ability of soft surfaces to enhance cell production. Toward immunotherapy, this result could be used to set a lower limit for the number of T_Eff_ cells that must be found or added to a starting population to ensure successful expansion. Considering that distinct T-cell phenotypes have been observed in mechanically diverse tissues [26], it is perhaps not surprising that different subtypes have different responses to substrate stiffness. However, while our results show that T_Eff_s determine population-level mechanosensing, they do not account for overall growth. Rather, naïve and/or memory T cells are responsible for the large number of resultant cells, requiring cell–cell communication with T_Eff_ cells by direct contact and/or paracrine signaling. Such mechanisms may include IL-2 secretion by CD8^+^ T cells, which drives the proliferation of memory T-cell types [27]. IL-2 supplementation of media culture also impacts the proliferation rate of the individual subsets differently [28]. In vitro, IL-2 secretion has been linked to mechanosensing as secretion levels exhibited a functional response to substrate stiffness [14], leading to a mechanism for population-level mechanosensing. Additionally, paracrine signaling of IFN-γ early in the immune response was found to have profound long-lasting effects on T-cell differentiation and clonal expansion; these interactions can be complex as IFN-γ antagonizes IL-2-driven signaling by modulating CD25 (IL-2 receptor) expression [29]. Mapping this network of intracellular signaling in the context of mechanosensing will be the subject of future studies. The techniques described here, including subtype depletion, coupled with more quantitative control over the frequency of different subtypes including naïve and/or regulatory T cells, matched with cytokine and transcriptomic profiling, will be central to these studies and potentially further enhance expansion [18,30].

One limitation of the current study is that the cohort of donors is of limited size, owing to the need to carry out multiple expansions for cells of each individual. Across 23 donors, there were data to reveal the dependencies on subtype frequency, which were subsequently confirmed using depletion studies. However, we do anticipate that the interactions between subtypes are complex, and further modified by additional, well-known sources of variation in immunity, most notably age and sex. Building off this initial proof-of-principle demonstration, future studies using the framework developed here will undoubtedly reveal new factors driving variation in human T-cell response.

Extending these studies beyond cells from healthy individuals is also needed to realize the full potential of the framework demonstrated here. Chronic cancer and the presence of a tumor microenvironment likely have impacts on T-cell response, including expansion and mechanosensing that are an added dimension beyond those applicable to healthy cells [31]. Indeed, T cells from CLL patients were found to have reduced proliferative potential [11]. Future studies could compare the functionality of T cells from cancer patients and healthy donors in systems that capture tumor microenvironments in vitro, using the framework developed here to more accurately account for donor variability and reveal new aspects of cancer progression and treatment [32,33].

In addition to cell–cell crosstalk and patient-derived T-cell samples, additional cell surface receptors could be incorporated into this mechanosensitive system. This report’s focus on CD3 and CD28 was driven by the widely adopted targeting of these receptors by cell activation systems. However, T-cell interactions with other cells are modulated by other ligands, including the integrin LFA-1 binding to its target ICAM-1 [34,35]. Complementary studies have demonstrated mechanosensing in this system [36,37], suggesting that the inclusion of ICAM-1 or other ligands could further enhance cell activation and expansion.

In summary, these results tame the variability in T-cell mechanosensing by showing that specific subtypes determine how a given population of cells will respond to activating substrate stiffness. This new framework can also be applied to other parameters, including additional mechanical features of biomaterials (e.g., viscoelasticity, porosity, topography, or inclusion of ICAM-1) and culture conditions (e.g., cytokine supplementation or oxygen availability) to more precisely improve cell expansion for immunotherapy. This framework can be used to identify differences in cell function beyond ex vivo expansion, including the impact on tumor growth and underlying functions needed in cancer therapy, such as the ability to recognize differences in local stiffness of the tumor microenvironment.

## Data Availability

The data that support the findings of this report are available from the authors upon reasonable request.

## Supplementary Materials

The following supporting information can be downloaded at https://www.mdpi.com/article/10.3390/cells14030203/s1: Figure S1: Gating strategy for a nine-color flow cytometry panel. Figure S2. Phenotypic composition of CD4+ and CD8+ T cells (n = 23). Figure S3. Pearson correlation coefficients between demographic information and phenotypic markers. Figure S4: Loadings of features to PC1 and PC2. Figure S5: Regression analysis investigating the linear relationship between principal components (PC) and MSF. Figure S6: Staining and subsequent flow cytometry of depletion study. Table S1: Demographic and phenotypic characteristics of donors and maximum doublings on hard and soft surfaces obtained from expansion experiments.
