# Supplementary material for: Taming Variability in T-Cell Mechanosensing
Source: Cells. 2025 Jan 30;14(3):203. doi: 10.3390/cells14030203 (PMC11817355; doi:10.3390/cells14030203)
Supplement: Supplementary file 1 [file cells-14-00203-s001.zip › cells-3410221-supplementary.pdf]

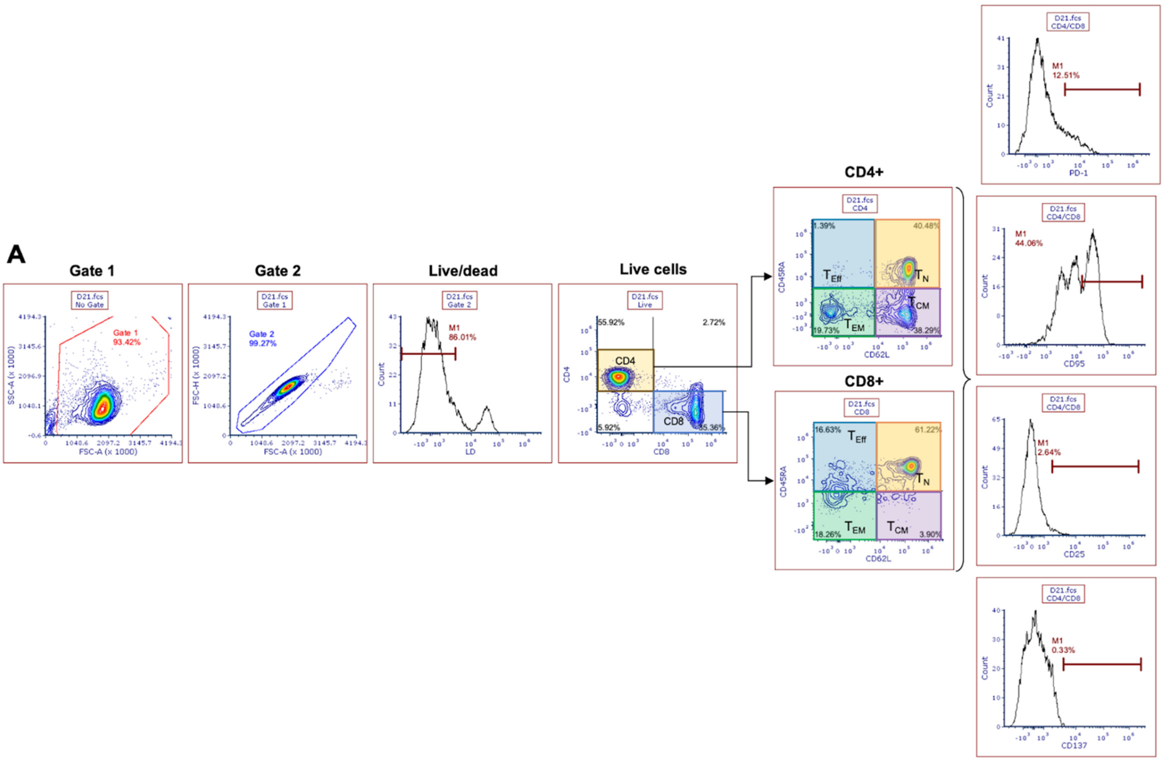

Figure S1. Gating strategy for a 9-color flow cytometry panel. Isolated T cells were stained for the cell surface markers CD3, CD4, CD8, CD45RA, CD62L, PD-1, CD95, CD25, CD137. A live/dead staining was used to identify viable cells. Sample gating strategy for a selected T cell blood sample is shown.

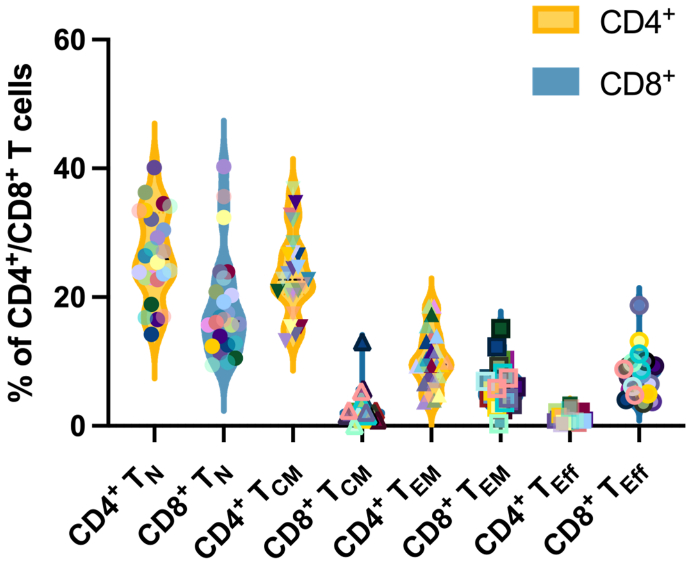

Figure S2. Phenotypic composition of CD4$^+$ and CD8$^+$ T cells (n=23). Symbols are color-coded by donor following Fig. 2D. T_{Eff} cells are mainly CD8$^+$.

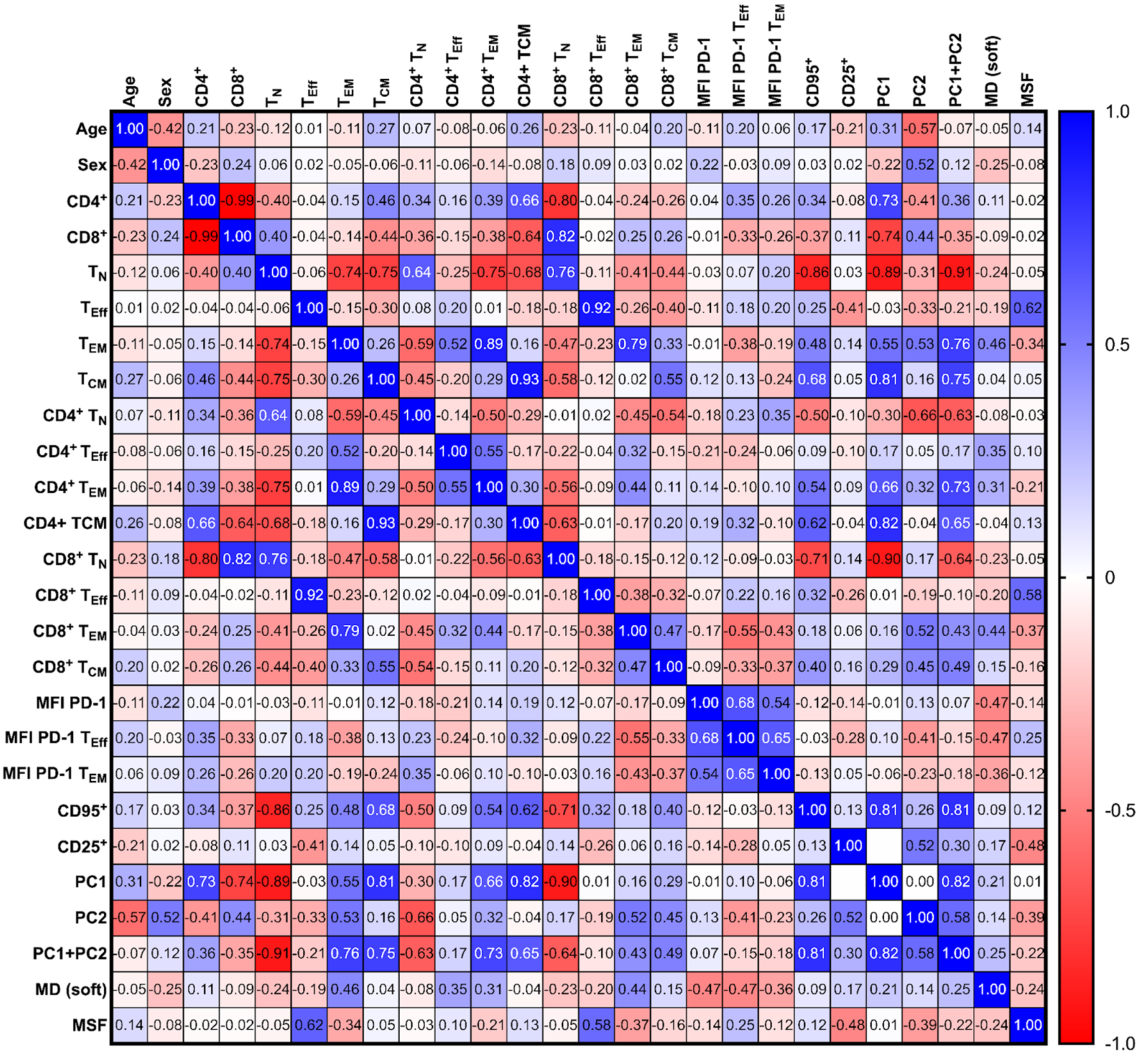

Figure S3. The Pearson correlation coefficients between demographic information and all phenotypic markers characterized in this study are shown above. MFI = median fluorescence intensity, MD = maximum doublings, MSF = mechanosensitivity factor.

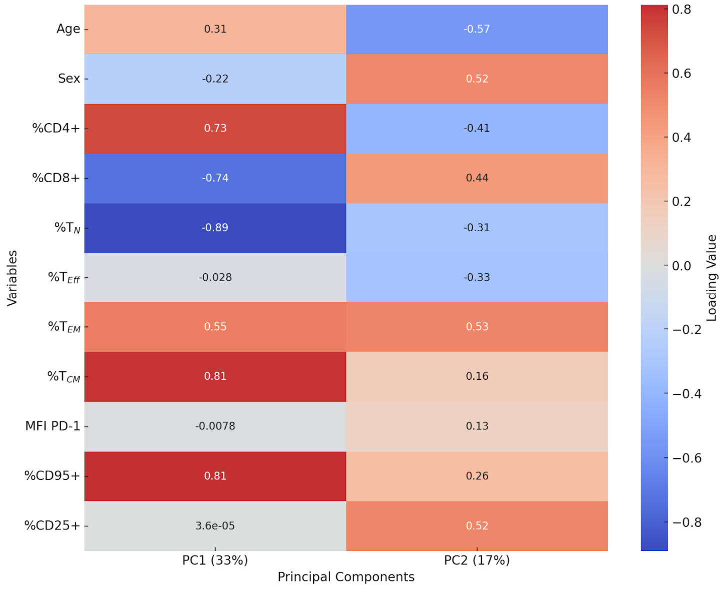

Figure S4. Loadings of features to PC1 and PC2. The loadings reveal which factors contribute to the variance of donor features and their T cell characteristics.

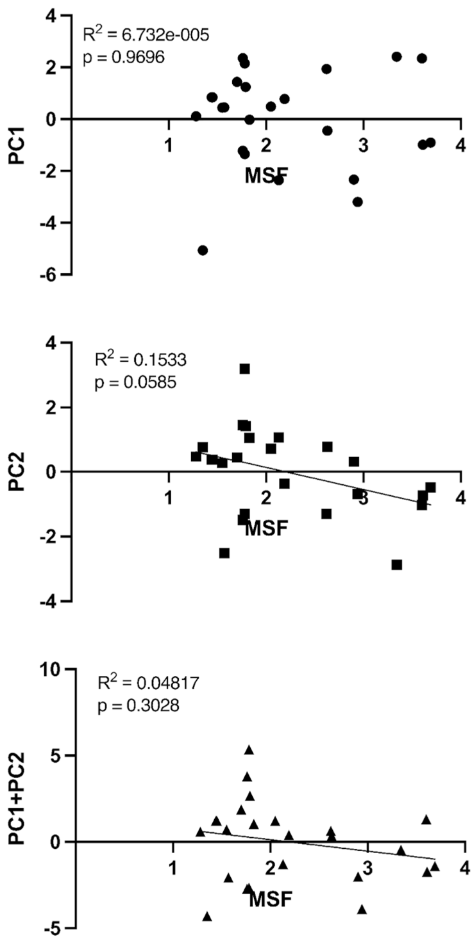

Figure S5. Regression analysis for investigating the linear relationship between principal components (PC) and MSF.

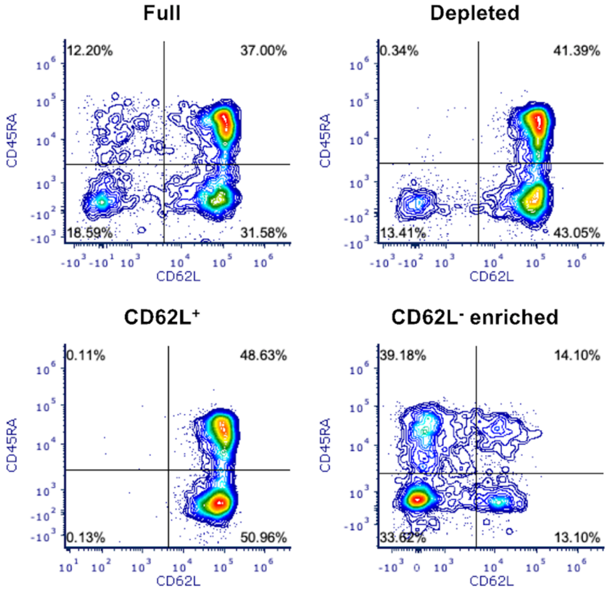

Figure S6. Staining and subsequent flow cytometry of depletion study. Sample flow cytometry dot plots for selected T cell blood samples are shown. Bead-based separation and reconstitution methods were used to modulate the subtype composition of starting cell populations (Full) by depleting T_{Eff} cells (Depleted). $CD62L^+$ cells contain $CD3^+$ T_N and T_{CM}; $CD62L^-$ cells contain mainly $CD3^+$ T_{Eff} and T_{EM}.

Table S1. Demographic and phenotypic characteristics of donors and maximum doublings on hard and soft surfaces obtained from expansion experiments. MSF was calculated as the ratio of the maximum doublings. MFI = median fluorescence intensity, MD = maximum doublings, MSF = mechanosensitivity factor.

Donor	Age	Sex	Race	%CD4$^+$	%T_N	%T_{Eff}	%T_{EM}	%T_{CM}	CD4$^+$ %T_N	CD4$^+$ %T_{Eff}	CD4$^+$ %T_{EM}	CD4$^+$ %T_{CM}	CD8$^+$ %T_N	CD8$^+$ %T_{Eff}	CD8$^+$ %T_{EM}	CD8$^+$ %T_{CM}	MFI PD-1	MFI PD-1 T_{Eff}	MFI PD-1 T_{EM}	%CD95$^+$	%CD25$^+$	CD4$^+$ %CD25$^+$	hard MD	soft MD	DY	MSF
D1	17.48	Female	Black	63.9	29.2	5.1	22.6	43.1	16.5	1.0	11.7	34.7	13.9	9.3	6.1	5.7	485.6	350.6	1318.1	69.7	3.0	4.5	3.5	6.3	6.0	1.78
D2	58.83	Female	White	45.2	53.7	6.2	12.5	27.6	17.0	1.4	4.9	21.9	35.6	4.8	7.4	5.4	233.6	202.5	1142.4	44.2	1.6	3.4	1.5	4.4	3.9	2.90
D3	34.00	Male	NA	65.1	39.0	8.0	29.4	23.5	23.0	2.3	18.7	21.0	15.7	5.6	10.1	2.3	486.0	571.8	3051.9	52.5	1.8	2.7	4.0	5.7	5.7	1.44
D3	34.00	Male	NA	65.1	39.0	8.0	29.4	23.5	23.0	2.3	18.7	21.0	15.7	5.6	10.1	2.3	486.0	571.8	3051.9	52.5	1.8	2.7	4.7	6.8	6.2	1.45
D4	29	Female	Hispanic	60.9	45.6	7.4	16.8	30.3	23.9	0.8	9.3	26.9	20.3	6.5	7.3	3.0	277.6	429.8	1859.3	58.4	2.0	3.2	2.4	4.4	3.9	1.83
D5	NA	Male	NA	46.2	60.1	15.0	8.3	16.7	25.4	0.5	4.9	15.4	32.4	13.1	2.9	1.0	823.0	1568.7	3150.4	46.6	1.7	3.6	1.2	3.5	4.3	2.94
D6	17	Female	NA	46.9	71.1	4.5	9.2	15.2	29.2	0.6	3.7	13.2	40.3	3.8	5.3	1.9	1412.5	1744.0	2583.0	24.4	1.5	3.2	3.7	5.0	3.5	1.35
D7	44	Female	NA	69.6	39.4	6.8	18.5	35.2	22.7	0.3	13.9	32.7	16.1	6.2	4.4	2.4	2789.3	4477.3	5216.6	55.2	1.7	2.4	1.8	3.1	3.9	1.70
D8	63	Male	White	66.2	38.4	10.6	18.2	32.8	27.5	0.6	9.5	28.4	9.9	8.6	8.2	3.8	397.2	1127.2	2049.6	64.1	1.8	2.4	2.0	5.3	3.3	2.62
D9	33	Female	White	71.3	46.9	7.2	15.9	29.9	33.5	1.7	9.5	26.6	12.4	5.0	5.4	2.9	375.8	549.3	2533.4	53.9	1.6	2.1	2.4	5.2	2.9	2.19
D10	50	Male	Asian/ Pacific Island	61.8	60.0	11.4	12.1	16.4	34.5	2.4	9.4	15.4	23.9	8.3	2.6	0.9	302.6	1678.9	3711.5	49.5	2.6	4.1	3.1	5.6	3.9	1.78
D11	30	Female	White	63.7	51.0	12.6	16.2	20.1	33.4	2.9	9.9	17.4	16.5	8.8	5.8	2.4	385.9	1392.1	2978.4	49.2	1.3	2.0	1.2	4.5	1.0	3.69
D12	30	Female	White	54.1	57.7	12.0	14.1	16.1	32.1	1.2	6.6	14.2	23.9	10.0	7.2	1.6	289.4	1247.0	4020.1	49.9	3.0	5.2	2.5	5.4		2.13
D13	26	Male	Asian	59.5	48.6	7.6	19.3	24.5	24.1	0.8	11.7	22.8	23.0	6.1	6.9	1.5	317.4	711.4	1449.2	53.6	2.6	4.4	1.7	4.5	4.0	2.63
D14	51	Male	White	73.4	52.2	8.9	12.4	26.3	40.1	0.8	8.0	24.4	11.4	7.6	3.3	1.6	219.1	929.9	3366.3	43.8	1.7	2.2	2.9	4.5	5.2	1.57
D15	33	Female	White	64.5	49.2	10.1	14.6	26.1	30.4	0.9	9.5	23.6	17.5	8.6	4.6	2.2	346.9	528.2	2305.6	63.8	2.3	3.5	3.2	4.2		1.28
D16	45	Male	Hispanic	67.1	30.9	15.1	19.9	34.0	16.8	2.6	15.6	32.0	12.8	11.1	3.6	1.6	574.9	1346.1	1887.7	66.9	1.3	1.9	1.6	5.6	5.2	3.60
D17	42	Female	Black	63.0	44.5	9.6	19.2	26.8	23.6	0.8	14.0	24.6	19.3	8.1	5.0	1.8	542.1	1039.0	3461.6	62.3	2.4	3.5	2.6	5.3	5.3	2.05
D18	52	Male	White	79.8	43.8	10.8	8.2	37.0	34.1	0.9	7.8	36.8	9.5	9.9	0.3	0.1	625.6	4469.7	3276.4	61.6	1.2	1.5	1.4	4.5	5.4	3.34

Donor	Age	Sex	Race	%CD4$^+$	%T_N	%T_{Eff}	%T_{EM}	%T_{CM}	CD4$^+$ %T_N	CD4$^+$ %T_{Eff}	CD4$^+$ %T_{EM}	CD4$^+$ %T_{CM}	CD8$^+$ %T_N	CD8$^+$ %T_{Eff}	CD8$^+$ %T_{EM}	CD8$^+$ %T_{CM}	MFI PD-1	MFI PD-1 T_{Eff}	MFI PD-1 T_{EM}	%CD95$^+$	%CD25$^+$	CD4$^+$ %CD25$^+$	hard MD	soft MD	DY	MSF
D19	31	Female	White	54.8	44.1	21.1	11.9	22.6	27.1	0.2	7.1	20.3	15.8	18.7	4.3	2.1	425.3	1621.4	2457.5	60.9	0.6	1.1	1.4	4.9	4.7	3.61
D20	48	Male	Asian	61.8	58.5	4.4	13.7	23.3	36.3	0.5	4.0	21.0	20.8	3.5	9.2	2.0	76.3	558.2	937.5	39.3	1.6	2.5	3.7	6.6	6.2	1.76
D21	49	Female	Asian	60.5	44.8	10.0	18.3	26.8	26.4	0.9	10.4	22.7	16.9	7.9	7.3	3.6	460.9	1576.1	3399.0	64.9	1.9	3.0	3.2	5.0	5.4	1.55
D22	52	Male	White	54.7	27.8	5.1	26.0	41.0	14.2	0.8	13.1	26.5	12.6	4.1	12.3	13.1	438.7	547.0	1635.1	73.9	2.0	3.4	3.0	5.4	5.9	1.76
D23	34	Female	Asian/Pacific Island	60.5	30.1	13.5	33.5	22.7	18.9	3.4	17.3	20.9	10.5	9.2	15.1	1.7	635.9	504.2	1595.7	65.0	1.4	2.2	3.0	5.3	5.3	1.79
